# Supplementary material for: Universal closed-tube barcoding for monitoring the shark and ray trade in megadiverse conservation hotspots
Source: iScience. 2023 Jun 7;26(7):107065. doi: 10.1016/j.isci.2023.107065 (PMC10300358; doi:10.1016/j.isci.2023.107065)
Supplement: Document S1. Figures S1–S7 and Tables S1–S7 [file mmc1.pdf]

iScience, Volume *26*

Supplemental information

Universal closed-tube barcoding for monitoring the shark and ray trade in megadiverse conservation hotspots

Andhika P. Prasetyo, Marine Cusa, Joanna M. Murray, Firdaus Agung, Efin Muttaqin, Stefano Mariani, and Allan D. McDevitt

Supplemental information for:

Universal closed-tube barcoding for monitoring the shark and ray trade in megadiverse conservation hotspots

Andhika P. Prasetyo[1,2,3*], Marine Cusa[1,4], Joanna M. Murray[5], Muh. Firdaus A. K. Kurniawan[6], Efin Muttaqin[7], Stefano Mariani[8†] and Allan D. McDevitt[1,9†*]

This file includes: Figures S1–S7 and Tables S1-S7

Supplementary figures

Figure S.1. The fluorescent signatures in BS1 of 14 shark species.

Figure S.2. The fluorescent signatures in BS2 of 14 shark species.

Figure S.3. The fluorescent signatures in BS1 of 14 ray species.

Figure S.4. The fluorescent signatures in BS2 of 14 ray species.

Figure S.5. Some species which have a hybridization problem in the BS1 region. Those species only have "TM" signature (the right-most valley in the BS1, labelled with a green color), TM corresponds to ThermaMark^{TM}, an internal marker for correction of artefactual temperature variation.

Figure S.6. Sampling locations across Java Island, Indonesia. Locations are labelled with long and short codes.

Figure S.7. A schematic description of the stages of this study which include (a) sample collection and preservation, (b) DNA extraction of tissue samples, (c-e) sample processing using the FASTFISH-ID workflow, (f) visualisation of the RT-PCR outputs and (g and h) species classification using deep learning.

Supplementary tables

Table S.1. Sample details used on the training datasets including Condition (processed/fresh), Part (of the animal), Species, ID (number), no. of replications and Sequencing technology used to identify the species.

Table S.2. Sample details used on the testing datasets including Condition (processed/fresh), Part (of the animal), Species, ID (number), no. of replications and Sequencing technology used to identify the species.

Table S.3. Variable importance in recognizing fluorescent signatures of species

Table S.4. Result of grid search in finding the best deep learning model

Table S.5. Assignment scoring of 28 species of sharks and rays

Table S.6. Initial value of hyper-parameters in searching for the best deep learning model using grid search method

Table S.7. Stopping criteria in searching the best deep learning model

Supplementary figures

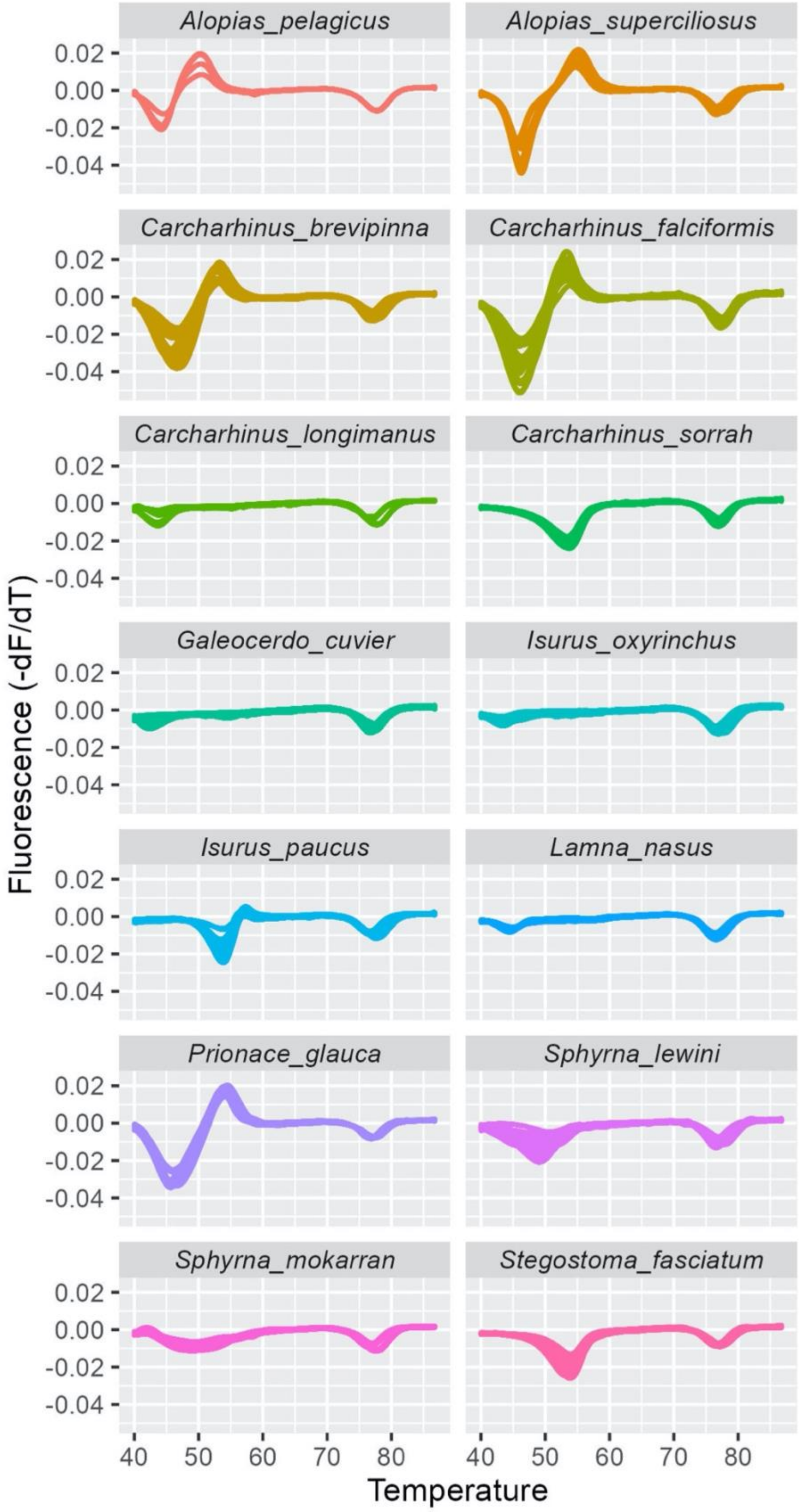

Figure S.1. The fluorescent signatures in BS1 of 14 shark species.

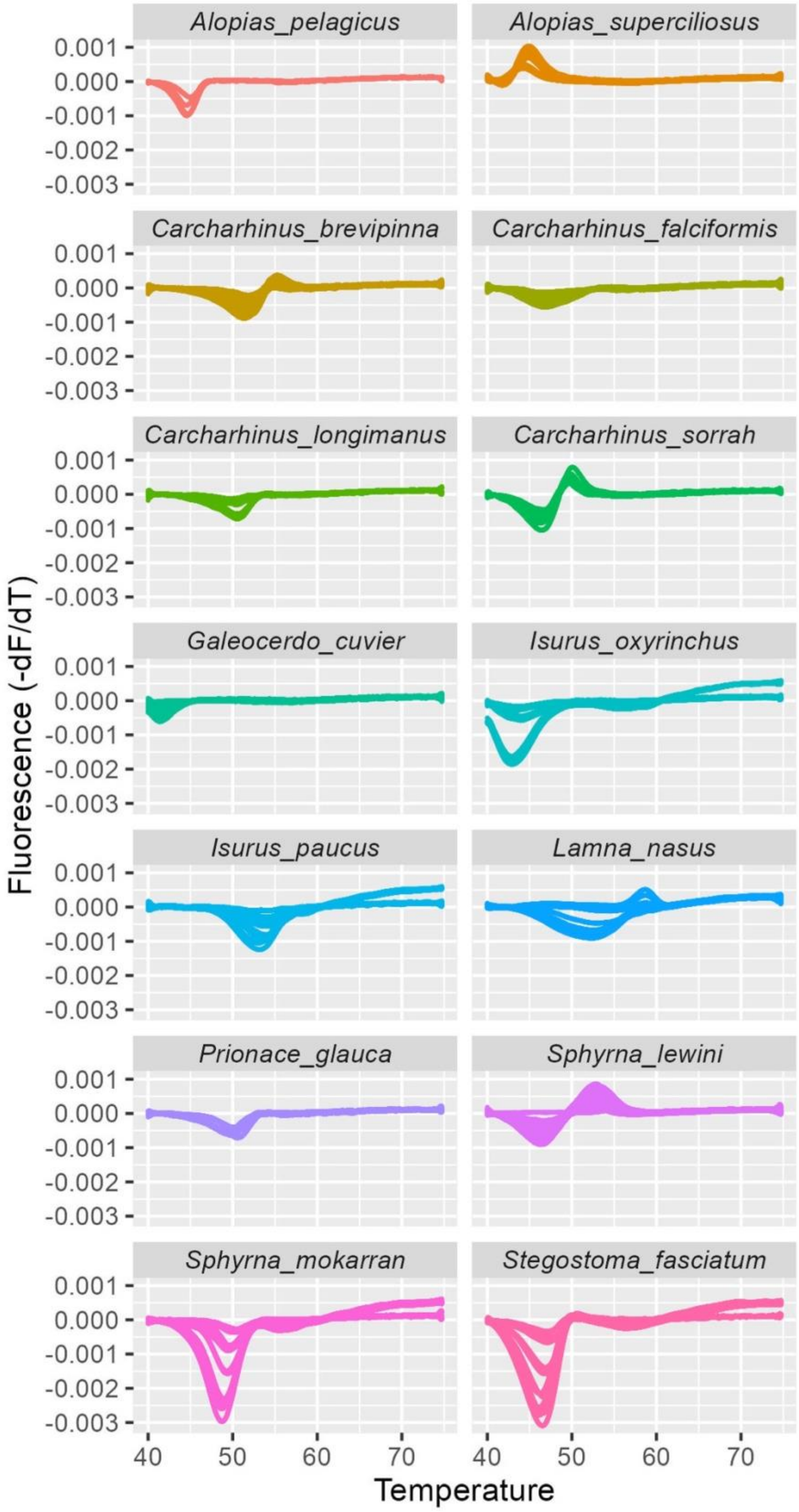

Figure S.2. The fluorescent signatures in BS2 of 14 shark species.

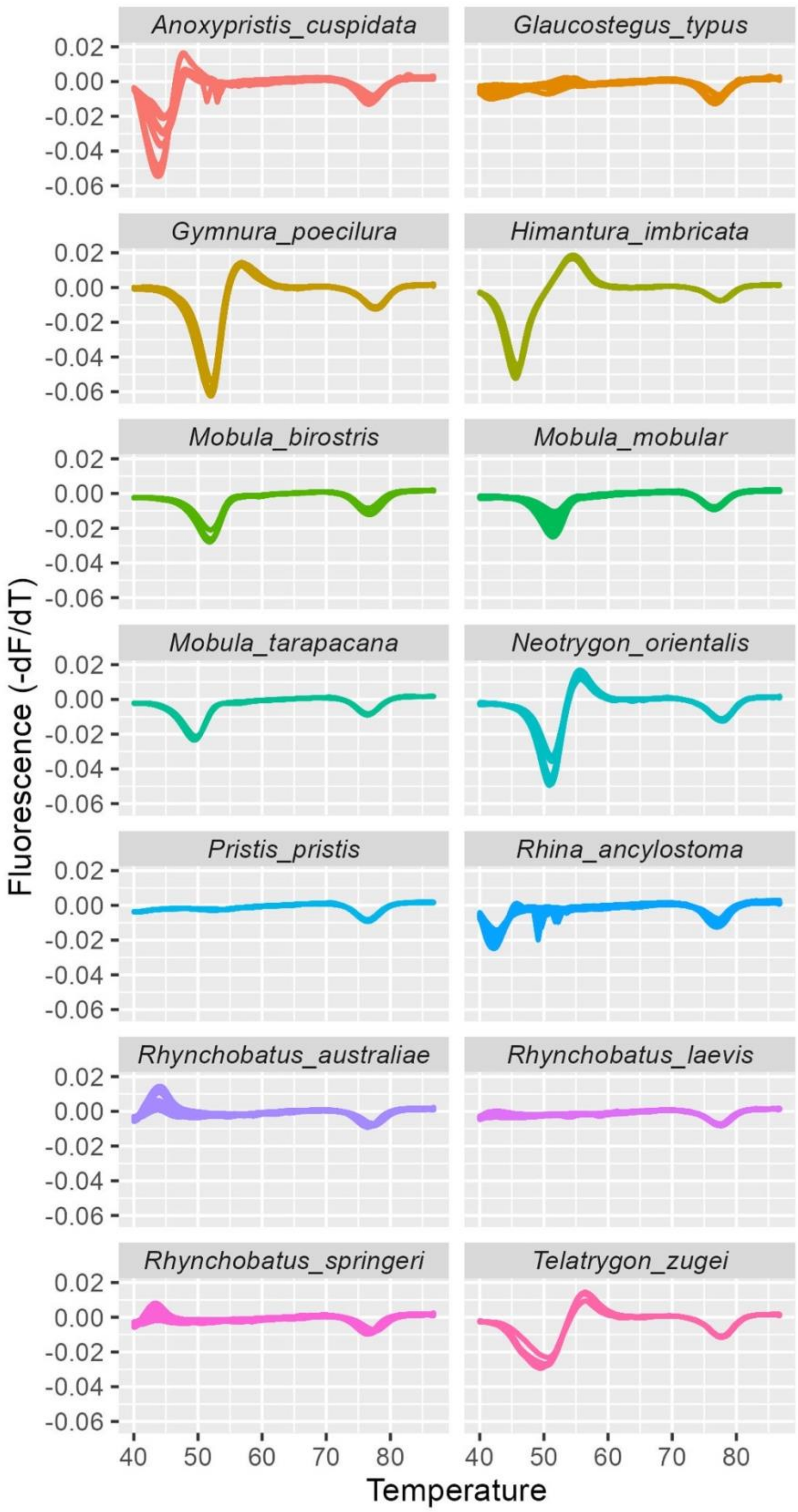

Figure S.3. The fluorescent signatures in BS1 of 14 ray species.

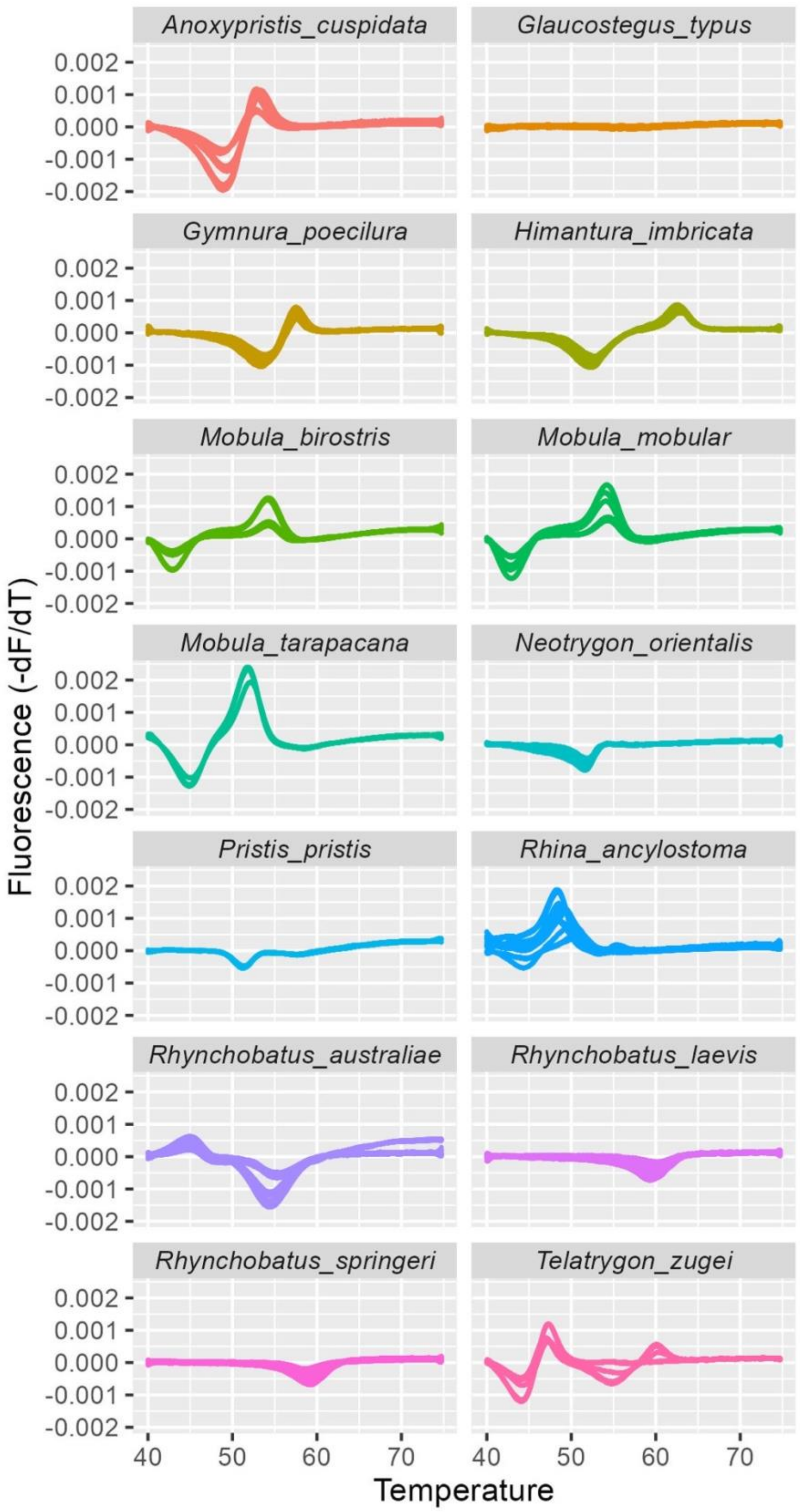

Figure S.4. The fluorescent signatures in BS2 of 14 ray species.

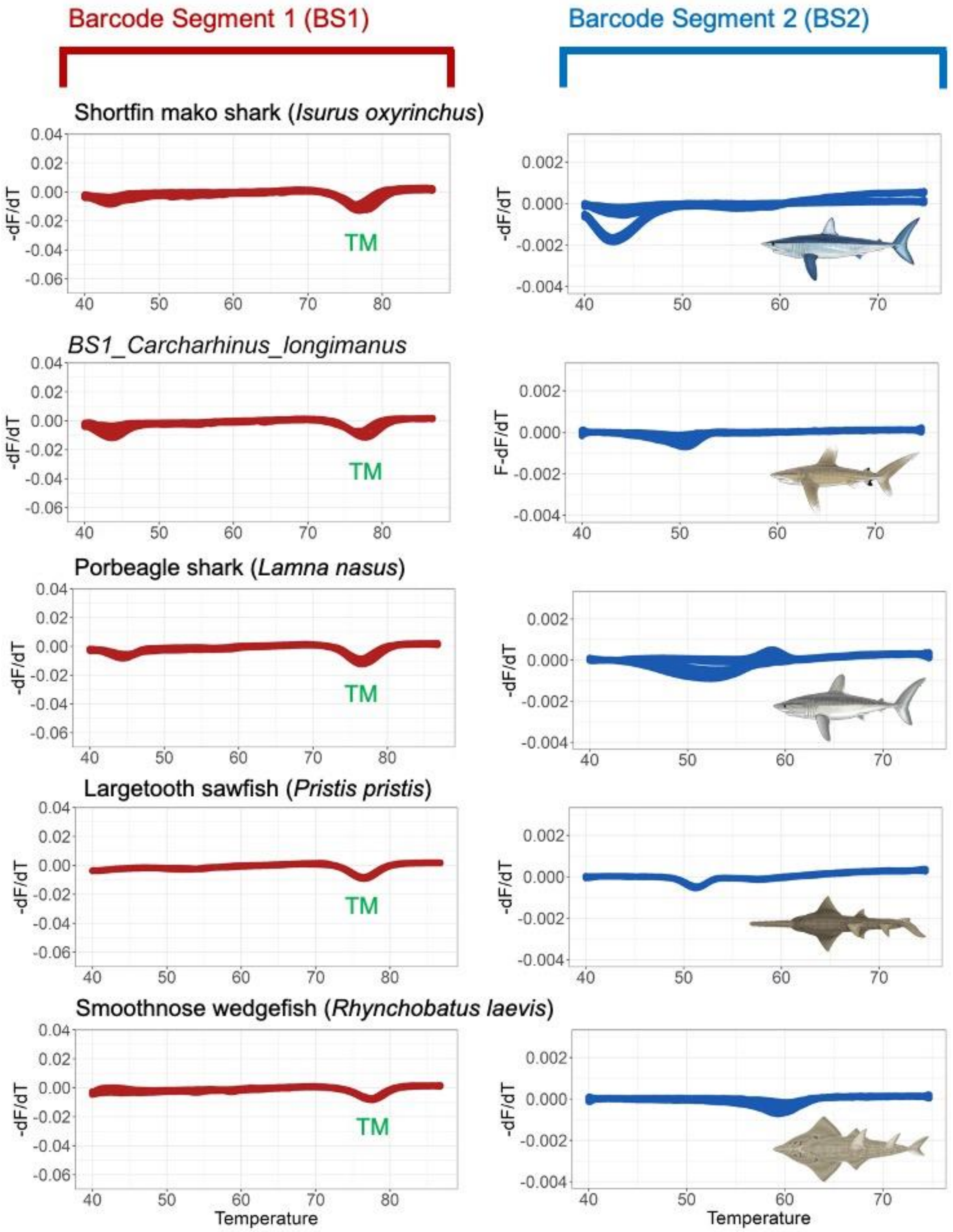

Figure S.5. Some species which have a hybridization problem in the BS1 region. Those species only have "TM" signature (the right-most valley in the BS1, labelled with a green color), TM corresponds to ThermaMark^{TM}, an internal marker for correction of artefactual temperature variation.

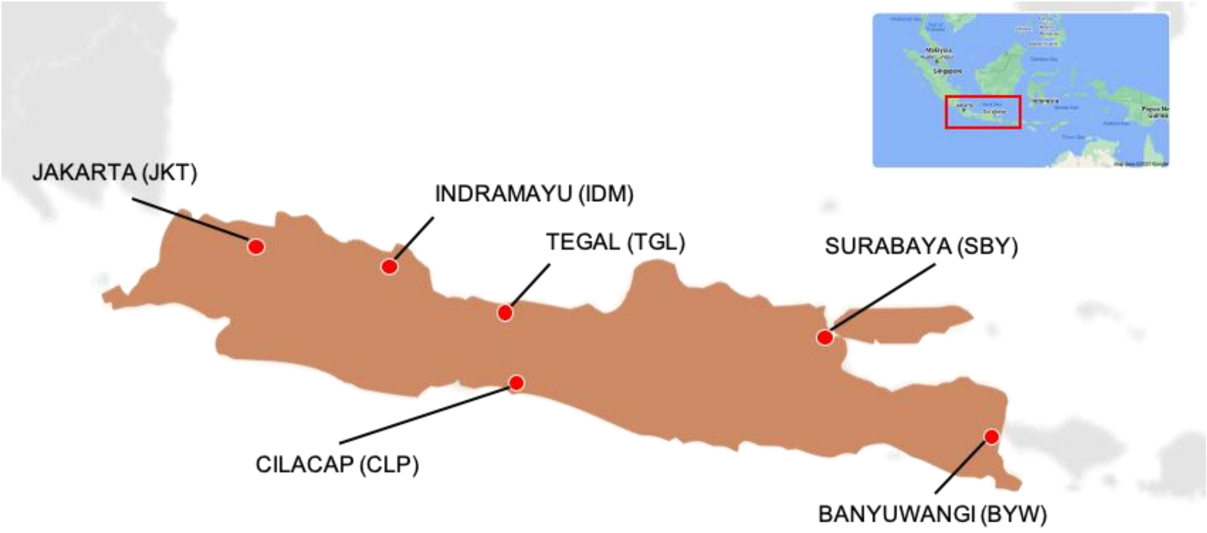

Figure S.6. Sampling locations across Java Island, Indonesia. Locations are labelled with long and short codes.

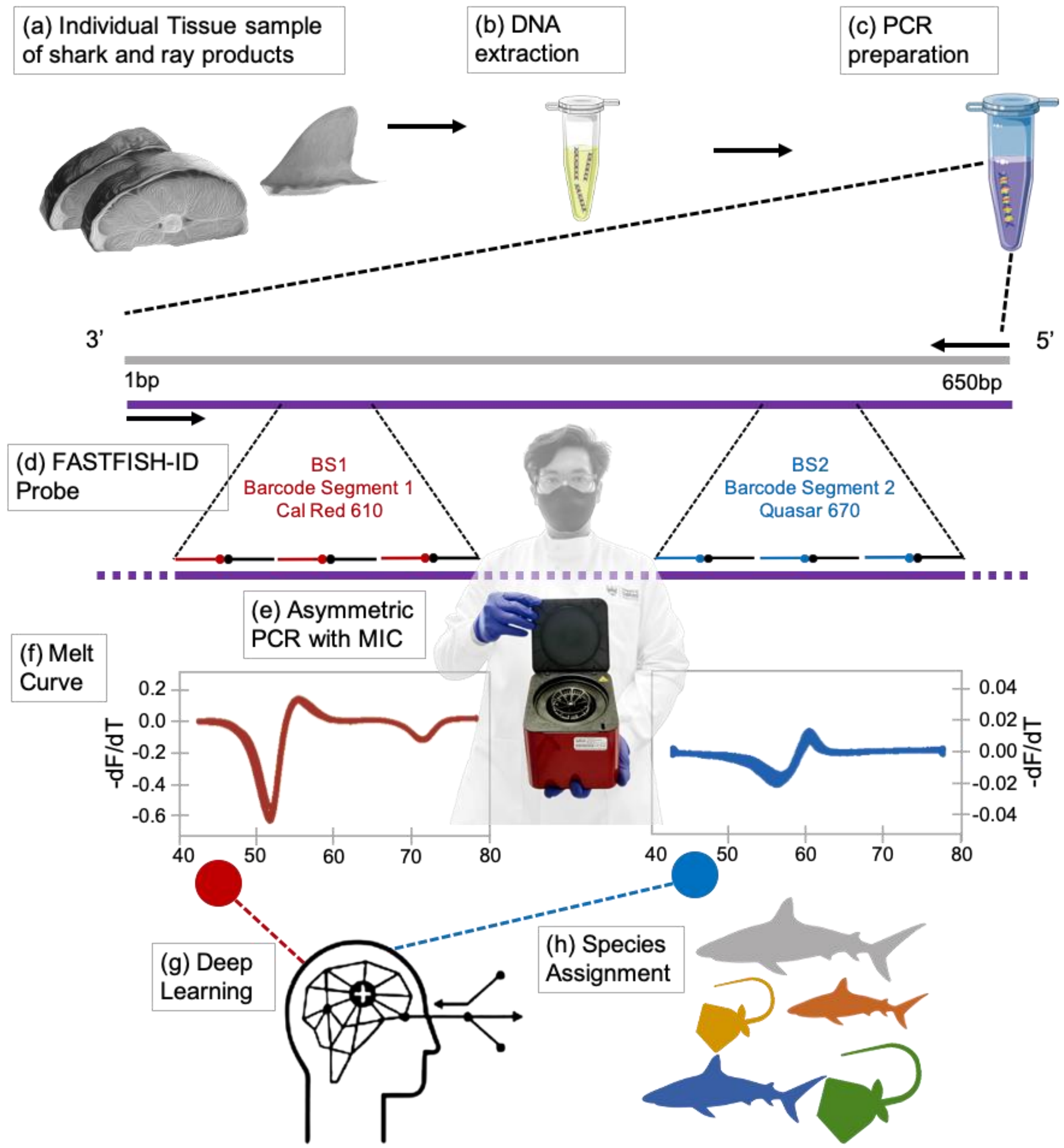

Figure S.7. A schematic description of the stages of this study which include a) sample collection and preservation, b) DNA extraction of tissue samples, c-e) sample processing using the FASTFISH-ID workflow, f) visualisation of the RT-PCR outputs and g-h) species classification using deep learning.

Supplementary tables

Table S.1. Sample details used on the training datasets including Condition (processed/fresh), Part (of the animal), Species, ID (number), no. of replications and Sequencing technology used to identify the species.

Condition	Part	Species	ID	Replication	Sequencing
Processed	Dried fin	*Alopias pelagicus*	340	3	Sanger ~650bp
Processed	Dried fin	*Alopias pelagicus*	341	2	Sanger ~650bp
Processed	Dried fin	*Alopias superciliosus*	54	3	HTB ~313bp
Processed	Dried fin	*Alopias superciliosus*	345	3	Sanger ~650bp
Processed	Dried fin	*Alopias superciliosus*	346	3	Sanger ~650bp
Processed	Salted meat	*Alopias superciliosus*	366	3	HTB ~313bp
Processed	Dried fin	*Alopias superciliosus*	431	2	Sanger ~650bp
Processed	Unidentified	*Alopias superciliosus*	530	3	HTB ~313bp
Processed	Rostrum	*Anoxypristis cuspidata*	9	4	Sanger ~650bp
Processed	Dried fin	*Anoxypristis cuspidata*	22	3	Sanger ~650bp
Processed	Unidentified	*Anoxypristis cuspidata*	536	3	HTB ~313bp
Processed	Rostrum	*Anoxypristis cuspidata*	490	2	Sanger ~650bp
Fresh	Trunk	*Carcharhinus brevipinna*	77	3	HTB ~313bp
Fresh	Trunk	*Carcharhinus brevipinna*	78	3	Sanger ~650bp
Fresh	Trunk	*Carcharhinus brevipinna*	86	2	Sanger ~650bp
Fresh	Finless	*Carcharhinus brevipinna*	123	3	HTB ~313bp
Fresh	Whole	*Carcharhinus brevipinna*	321	1	Sanger ~650bp
Fresh	Whole	*Carcharhinus brevipinna*	323	3	Sanger ~650bp
Fresh	Whole	*Carcharhinus brevipinna*	324	3	Sanger ~650bp
Fresh	Whole	*Carcharhinus brevipinna*	334	3	Sanger ~650bp

Condition	Part	Species	ID	Replication	Sequencing
Fresh	Whole	*Carcharhinus brevipinna*	475	1	Sanger ~650bp
Fresh	Trunk	*Carcharhinus falciformis*	3	3	HTB ~313bp
Fresh	Trunk	*Carcharhinus falciformis*	4	3	HTB ~313bp
Fresh	Trunk	*Carcharhinus falciformis*	5	3	HTB ~313bp
Fresh	Trunk	*Carcharhinus falciformis*	6	3	HTB ~313bp
Fresh	Trunk	*Carcharhinus falciformis*	7	3	HTB ~313bp
Fresh	Trunk	*Carcharhinus falciformis*	43	3	Sanger ~650bp
Fresh	Whole	*Carcharhinus falciformis*	285	3	Sanger ~650bp
Fresh	Whole	*Carcharhinus falciformis*	293	2	Sanger ~650bp
Fresh	Whole	*Carcharhinus falciformis*	294X	3	Sanger ~650bp
Processed	Dried fin	*Carcharhinus longimanus*	25	3	Sanger ~650bp
Processed	Dried fin	*Carcharhinus longimanus*	53	3	Sanger ~650bp
Processed	Dried fin	*Carcharhinus longimanus*	342	2	Sanger ~650bp
Fresh	Trunk	*Carcharhinus sorrah*	29	3	HTB ~313bp
Fresh	Trunk	*Carcharhinus sorrah*	46	3	HTB ~313bp
Fresh	Whole	*Carcharhinus sorrah*	185	3	Sanger ~650bp
Fresh	Whole	*Carcharhinus sorrah*	319	1	Sanger ~650bp
Fresh	Whole	*Galeocerdo cuvier*	178	3	HTB ~313bp
Fresh	Whole	*Galeocerdo cuvier*	363	3	HTB ~313bp
Fresh	Fin	*Galeocerdo cuvier*	456	1	Sanger ~650bp
Processed	Dried fin	*Galeocerdo cuvier*	354	3	Sanger ~650bp
Processed	Dried fin	*Galeocerdo cuvier*	435	3	Sanger ~650bp
Processed	Dried fin	*Galeocerdo cuvier*	436	3	Sanger ~650bp
**Condition**	**Part**	**Species**	**ID**	**Replication**	**Sequencing**
Processed	Dried fin	*Galeocerdo cuvier*	437	3	Sanger ~650bp

Processed	Teeth	*Galeocerdo cuvier*	439	3	Sanger ~650bp
Fresh	Whole	*Glaucostegus typus*	212	3	HTB ~313bp
Fresh	Whole	*Glaucostegus typus*	268	5	Sanger ~650bp
Processed	Dried fin	*Glaucostegus typus*	11	3	HTB ~313bp
Processed	Dried skin	*Glaucostegus typus*	196	3	HTB ~313bp
Fresh	Whole	*Gymnura poecilura*	90	3	Sanger ~650bp
Fresh	Whole	*Gymnura poecilura*	91	3	Sanger ~650bp
Fresh	Whole	*Gymnura poecilura*	92	3	Sanger ~650bp
Fresh	Whole	*Himantura imbricata*	296	3	Sanger ~650bp
Fresh	Whole	*Himantura imbricata*	297	2	Sanger ~650bp
Processed	Dried fin	*Isurus oxyrinchus*	50	3	Sanger ~650bp
Processed	Dried fin	*Isurus oxyrinchus*	343	3	Sanger ~650bp
Processed	Dried fin	*Isurus oxyrinchus*	344	2	Sanger ~650bp
Processed	Dried fin	*Isurus oxyrinchus*	384	3	HTB ~313bp
Processed	Dried fin	*Isurus oxyrinchus*	421	3	HTB ~313bp
Processed	Unidentified	*Isurus oxyrinchus*	519	3	Sanger ~650bp
Processed	Unidentified	*Isurus oxyrinchus*	521	2	Sanger ~650bp
Processed	Dried fin	*Isurus paucus*	20	3	Sanger ~650bp
Processed	Dried fin	*Isurus paucus*	52	3	Sanger ~650bp
Processed	Dried fin	*Isurus paucus*	338	3	Sanger ~650bp
Processed	Dried fin	*Isurus paucus*	339	2	Sanger ~650bp
Processed	Unidentified	*Isurus paucus*	528	3	HTB ~313bp
Processed	Unidentified	*Isurus paucus*	533	3	HTB ~313bp
Processed	Dried fin	*Lamna nasus*	24	3	HTB ~313bp
Processed	Dried fin	*Lamna nasus*	505	3	HTB ~313bp

**Condition**	**Part**	**Species**	**ID**	**Replication**	**Sequencing**
Processed	Dried fin	*Lamna nasus*	506	3	HTB ~313bp
Processed	Unidentified	*Lamna nasus*	527	3	HTB ~313bp

Processed	Salted meat	*Mobula birostris*	370	3	HTB ~313bp
Processed	Gill racker	*Mobula birostris*	412	3	HTB ~313bp
Processed	Gill racker	*Mobula mobular*	448	3	HTB ~313bp
Processed	Gill racker	*Mobula mobular*	449	3	HTB ~313bp
Processed	Gill racker	*Mobula mobular*	450	3	HTB ~313bp
Processed	Gill racker	*Mobula mobular*	451	3	HTB ~313bp
Processed	Cartillage	*Mobula tarapacana*	12	3	HTB ~313bp
Fresh	Whole	*Neotrygon orientalis*	240	3	Sanger ~650bp
Fresh	Whole	*Neotrygon orientalis*	241	1	Sanger ~650bp
Fresh	Whole	*Neotrygon orientalis*	244	3	Sanger ~650bp
Fresh	Trunk	*Prionace glauca*	413	3	Sanger ~650bp
Processed	Dried fin	*Prionace glauca*	355	3	Sanger ~650bp
Processed	Dried fin	*Prionace glauca*	356	3	Sanger ~650bp
Processed	Unidentified	*Pristis pristis*	550	3	HTB ~313bp
Fresh	Whole	*Rhina ancylostoma*	276	3	Sanger ~650bp
Fresh	Whole	*Rhina ancylostoma*	211	3	Sanger ~650bp
Processed	Dried fin	*Rhina ancylostoma*	27	3	Sanger ~650bp
Processed	Dried skin	*Rhina ancylostoma*	48	4	Sanger ~650bp
Processed	Meat	*Rhina ancylostoma*	247	3	HTB ~313bp
Fresh	Whole	*Rhynchobatus australiae*	101	3	Sanger ~650bp
Fresh	Finless	*Rhynchobatus australiae*	175	3	Sanger ~650bp
Fresh	Whole	*Rhynchobatus australiae*	213	2	Sanger ~650bp
Fresh	Whole	*Rhynchobatus australiae*	229	1	HTB ~313bp
Fresh	Whole	*Rhynchobatus australiae*	259	1	HTB ~313bp
Fresh	Whole	*Rhynchobatus australiae*	279	3	Sanger ~650bp
Processed	Dried fin	*Rhynchobatus australiae*	424	3	HTB ~313bp

**Condition**	**Part**	**Species**	**ID**	**Replication**	**Sequencing**
Fresh	Whole	*Rhynchobatus laevis*	35	3	Sanger ~650bp
Fresh	Whole	*Rhynchobatus laevis*	151	3	Sanger ~650bp

Fresh	Whole	*Rhynchobatus laevis*	152	3	Sanger ~650bp
Fresh	Finless	*Rhynchobatus laevis*	177	3	Sanger ~650bp
Fresh	Whole	*Rhynchobatus springeri*	189	3	Sanger ~650bp
Fresh	Whole	*Rhynchobatus springeri*	214	3	Sanger ~650bp
Fresh	Whole	*Rhynchobatus springeri*	215	1	HTB ~313bp
Fresh	Whole	*Rhynchobatus springeri*	221	3	Sanger ~650bp
Fresh	Whole	*Rhynchobatus springeri*	224	1	Sanger ~650bp
Fresh	Whole	*Rhynchobatus springeri*	226	1	Sanger ~650bp
Fresh	Whole	*Rhynchobatus springeri*	258	3	Sanger ~650bp
Fresh	Whole	*Rhynchobatus springeri*	274	3	Sanger ~650bp
Fresh	Finless	*Sphyrna lewini*	112	3	Sanger ~650bp
Fresh	Whole	*Sphyrna lewini*	115	3	Sanger ~650bp
Fresh	Whole	*Sphyrna lewini*	121	3	Sanger ~650bp
Fresh	Finless	*Sphyrna lewini*	122	3	HTB ~313bp
Fresh	Finless	*Sphyrna lewini*	126	3	Sanger ~650bp
Fresh	Whole	*Sphyrna lewini*	476	3	Sanger ~650bp
Processed	Dried fin	*Sphyrna lewini*	16	3	HTB ~313bp
Processed	Dried fin	*Sphyrna lewini*	426	1	Sanger ~650bp
Fresh	Finless	*Sphyrna mokarran*	113	3	Sanger ~650bp
Processed	Cartillage	*Sphyrna mokarran*	13	3	HTB ~313bp
Processed	Dried fin	*Sphyrna mokarran*	21	3	Sanger ~650bp
Processed	Dried skin	*Sphyrna mokarran*	197	3	HTB ~313bp
Processed	Salted meat	*Sphyrna mokarran*	367	3	HTB ~313bp
Processed	Dried fin	*Sphyrna mokarran*	418	2	Sanger ~650bp
Fresh	Whole	*Stegostoma fasciatum*	133	3	HTB ~313bp
Fresh	Trunk	*Stegostoma fasciatum*	179	3	HTB ~313bp
**Condition**	**Part**	**Species**	**ID**	**Replication**	**Sequencing**
Fresh	Trunk	*Stegostoma fasciatum*	180	3	HTB ~313bp
Fresh	Trunk	*Stegostoma fasciatum*	181	3	Sanger ~650bp

Processed	Dried fin	*Stegostoma fasciatum*	583	1	Sanger ~650bp
Fresh	Whole	*Telatrygon zugei*	198	3	Sanger ~650bp
Fresh	Whole	*Telatrygon zugei*	245	2	Sanger ~650bp

Table S.2. Sample details used on the testing datasets including Condition (processed/fresh), Part (of the animal), Species, ID (number), no. of replications and Sequencing technology used to identify the species.

Condition	Part	Species	ID	Replication	Sequencing
Processed	Dried fin	*Alopias pelagicus*	340	1	Sanger ~650bp
Processed	Dried fin	*Alopias superciliosus*	431	1	Sanger ~650bp
Processed	Unidentified	*Alopias superciliosus*	535	1	HTB ~313bp
Processed	Unidentified	*Anoxypristis cuspidata*	536	1	HTB ~313bp
Fresh	Whole	*Carcharhinus brevipinna*	317	1	HTB ~313bp
Fresh	Whole	*Carcharhinus brevipinna*	321	1	Sanger ~650bp
Fresh	Whole	*Carcharhinus brevipinna*	322	1	HTB ~313bp
Fresh	Whole	*Carcharhinus brevipinna*	326	1	HTB ~313bp
Fresh	Whole	*Carcharhinus brevipinna*	475	1	Sanger ~650bp
Fresh	Trunk	*Carcharhinus falciformis*	43	1	Sanger ~650bp
Fresh	Trunk	*Carcharhinus falciformis*	4	1	HTB ~313bp
Fresh	Trunk	*Carcharhinus falciformis*	19	1	HTB ~313bp
Fresh	Trunk	*Carcharhinus falciformis*	58	1	HTB ~313bp
Processed	Dried fin	*Carcharhinus longimanus*	342	1	Sanger ~650bp
Processed	Unidentified	*Carcharhinus longimanus*	522	1	HTB ~313bp
Processed	Unidentified	*Carcharhinus longimanus*	523	1	HTB ~313bp
Processed	Unidentified	*Carcharhinus longimanus*	524	1	HTB ~313bp
Fresh	Whole	*Carcharhinus sorrah*	304	1	Sanger ~650bp
Processed	Oil	*Galeocerdo cuvier*	396	1	HTB ~313bp
Processed	Dried fin	*Galeocerdo cuvier*	432	1	HTB ~313bp
Processed	Dried fin	*Galeocerdo cuvier*	433	1	HTB ~313bp
Processed	Dried fin	*Galeocerdo cuvier*	434	1	HTB ~313bp

Condition	Part	Species	ID	Replication	Sequencing
Processed	Dried skin	*Galeocerdo cuvier*	441	1	Sanger ~650bp
Fresh	Whole	*Glaucostegus typus*	272	1	Sanger ~650bp
Fresh	Whole	*Glaucostegus typus*	275	1	HTB ~313bp
Processed	Dried fin	*Glaucostegus typus*	422	1	HTB ~313bp
Processed	Dried fin	*Glaucostegus typus*	428	1	HTB ~313bp
Processed	Unidentified	*Glaucostegus typus*	537	1	HTB ~313bp
Fresh	Whole	*Gymnura poecilura*	88	1	Sanger ~650bp
Fresh	Whole	*Gymnura poecilura*	89	1	Sanger ~650bp
Fresh	Whole	*Himantura imbricata*	297	1	Sanger ~650bp
Processed	Dried fin	*Isurus oxyrinchus*	344	1	Sanger ~650bp
Processed	Unidentified	*Isurus oxyrinchus*	531	1	HTB ~313bp
Processed	Dried fin	*Isurus paucus*	339	1	Sanger ~650bp
Processed	Dried fin	*Lamna nasus*	24	1	HTB ~313bp
Processed	Unidentified	*Lamna nasus*	529	1	HTB ~313bp
Processed	Salted meat	*Mobula birostris*	370	1	HTB ~313bp
Processed	Gill racker	*Mobula mobular*	451	1	HTB ~313bp
Processed	Cartillage	*Mobula tarapacana*	12	1	HTB ~313bp
Fresh	Whole	*Neotrygon orientalis*	242	1	Sanger ~650bp
Fresh	Trunk	*Prionace glauca*	414	1	HTB ~313bp
Fresh	Trunk	*Prionace glauca*	416	1	Sanger ~650bp
Fresh	Trunk	*Prionace glauca*	417	1	HTB ~313bp
Processed	Dried fin unskin	*Prionace glauca*	399	1	HTB ~313bp
Processed	Dried fin	*Prionace glauca*	410	1	HTB ~313bp
Processed	Unidentified	*Pristis pristis*	550	1	HTB ~313bp
Processed	Dried fin	*Rhina ancylostoma*	14	1	Sanger ~650bp
Processed	Dried skin	*Rhina ancylostoma*	48	1	Sanger ~650bp
Fresh	Whole	*Rhynchobatus australiae*	213	1	Sanger ~650bp
Fresh	Whole	*Rhynchobatus laevis*	39	1	Sanger ~650bp
**Condition**	**Part**	**Species**	**ID**	**Replication**	**Sequencing**

Fresh	Finless	*Rhynchobatus laevis*	176	1	HTB ~313bp
Processed	Unidentified	*Rhynchobatus laevis*	534	1	HTB ~313bp
Fresh	Whole	*Rhynchobatus springeri*	217	1	HTB ~313bp
Fresh	Whole	*Rhynchobatus springeri*	224	1	Sanger ~650bp
Fresh	Whole	*Rhynchobatus springeri*	225	1	Sanger ~650bp
Fresh	Whole	*Rhynchobatus springeri*	226	1	Sanger ~650bp
Fresh	Whole	*Rhynchobatus springeri*	223B	1	Sanger ~650bp
Fresh	Finless	*Sphyrna lewini*	125	1	HTB ~313bp
Fresh	Trunk	*Sphyrna lewini*	155	1	HTB ~313bp
Fresh	Trunk	*Sphyrna lewini*	156	1	HTB ~313bp
Fresh	Whole	*Sphyrna lewini*	160	1	HTB ~313bp
Fresh	Whole	*Sphyrna lewini*	234	1	Sanger ~650bp
Processed	Dried fin	*Sphyrna lewini*	419	1	Sanger ~650bp
Processed	Dried fin	*Sphyrna lewini*	426	1	Sanger ~650bp
Processed	Dried fin	*Sphyrna mokarran*	418	1	Sanger ~650bp
Processed	Dried fin	*Sphyrna mokarran*	420	1	HTB ~313bp
Processed	Dried skin	*Stegostoma fasciatum*	195	1	HTB ~313bp
Fresh	Whole	*Telatrygon zugei*	245	1	Sanger ~650bp

Table S.3. Variable importance in recognizing fluorescent signatures of species

Barcode segment	Variable	Relative importance	Scaled importance	Percentage
BS1	C5	1	1	1.87E-04
BS1	C13	0.97	0.97	1.81E-04
BS1	C15	0.96	0.96	1.80E-04
BS1	C17	0.97	0.97	1.82E-04
…	…	…	…	…
BS1	C2635	0.53	0.53	9.90E-05
BS2	C4678	0.98	0.98	1.82E-04
BS2	C6741	0.52	0.52	9.81E-05
BS2	C6747	0.53	0.53	9.92E-05
BS2	C6748	0.53	0.53	9.91E-05
BS2	C6750	0.53	0.53	9.90E-05

Table S.4. Result of grid search in finding the best deep learning model

No	Model ID	Accuracy	Activation function	Epochs	Epsilon	Hidden layers	Input dropout ratio	L1	L2	Max w2	Rho
1	dl_grid_model_17	0.98	RectifierWithDropout	500	1.00E-08	[500, 500, 500]	0.2	0	0.0001	1000	0.9
2	dl_grid_model_170	0.98	Maxout	300	1.00E-06	[500, 500, 500]	0.2	0	0	100	0.9
3	dl_grid_model_7	0.98	MaxoutWithDropout	500	1.00E-06	[100, 100, 100]	0	0	0.0001	100	0.95
4	dl_grid_model_104	0.97	Tanh	500	1.00E-10	[100, 100, 100]	0.2	0	0	10	0.95
5	dl_grid_model_107	0.97	TanhWithDropout	300	1.00E-06	[500, 500, 500]	0	1E-05	1E-05	1000	0.95
...	...	...	...	...	...	...	...	...	...	...	...
7	dl_grid_model_32	0.01	Rectifier	300	1.00E-04	[100, 100, 100]	0.1	0	0	10	1
8	dl_grid_model_195	0.00	Rectifier	100	1.00E-04	[500, 500, 500]	0	0	0	10	1
9	dl_grid_model_247	0.00	RectifierWithDropout	200	1.00E-06	[500, 500, 500]	0	0	0	1000	1
10	dl_grid_model_260	0.00	RectifierWithDropout	300	1.00E-04	[200, 200, 200]	0	0	1E-05	100	0.95
11	dl_grid_model_66	0.00	RectifierWithDropout	50	1.00E-04	[200, 200, 200]	0	0	0.0001	100	0.95

Table S.5. Assignment scoring of 28 species of sharks and rays

No.	Actual	Prediction		SCORE	Alopias_pelagicus	Alopias_superciliosus	Anoxypristis_cuspidata	Carcharhinus_brevipinna	Carcharhinus_falciformis	Carcharhinus_longimanus	Carcharhinus_sorrah	Galeocerdo_cuvier	Glaucostegus_typus	Gymnura_poecilura	Himantura_imbricata	Isurus_oxyrinchus	Isurus_paucus	Lamna_nasus	Mobula_birostris	Mobula_mobular	Mobula_tarapacana	Neotrygon_orientalis	Prionace_glauca	Pristis_pristis	Rhina_ancylostoma	Rhynchobatus_australiae	Rhynchobatus_laevis	Rhynchobatus_springeri	Sphyrna_lewini	Sphyrna_mokarran	Stegostoma_fasciatum	Telatrygon_zugei
1	Glaucostegus_typus	Glaucostegus_typus	Match	1.000	0.000	0.000	0.000	0.000	0.000	0.000	0.000	0.000	1.000	0.000	0.000	0.000	0.000	0.000	0.000	0.000	0.000	0.000	0.000	0.000	0.000	0.000	0.000	0.000	0.000	0.000	0.000	0.000
2	Rhina_ancylostoma	Rhina_ancylostoma	Match	1.000	0.000	0.000	0.000	0.000	0.000	0.000	0.000	0.000	0.000	0.000	0.000	0.000	0.000	0.000	0.000	0.000	0.000	0.000	0.000	0.000	1.000	0.000	0.000	0.000	0.000	0.000	0.000	0.000
3	Rhynchobatus_laevis	Rhynchobatus_laevis	Match	0.545	0.004	0.001	0.000	0.000	0.001	0.000	0.001	0.319	0.102	0.000	0.000	0.000	0.000	0.000	0.000	0.000	0.001	0.000	0.002	0.000	0.002	0.001	0.545	0.000	0.019	0.000	0.000	0.000
4	Rhynchobatus_springer	Rhynchobatus_springer	Match	0.999	0.000	0.000	0.000	0.000	0.000	0.000	0.000	0.000	0.000	0.000	0.000	0.000	0.000	0.000	0.000	0.000	0.000	0.000	0.000	0.000	0.000	0.000	0.001	0.999	0.000	0.000	0.000	0.000
5	Rhynchobatus_springer	Rhynchobatus_springer	Match	1.000	0.000	0.000	0.000	0.000	0.000	0.000	0.000	0.000	0.000	0.000	0.000	0.000	0.000	0.000	0.000	0.000	0.000	0.000	0.000	0.000	0.000	0.000	0.000	1.000	0.000	0.000	0.000	0.000
6	Gymnura_poecilura	Gymnura_poecilura	Match	1.000	0.000	0.000	0.000	0.000	0.000	0.000	0.000	0.000	0.000	1.000	0.000	0.000	0.000	0.000	0.000	0.000	0.000	0.000	0.000	0.000	0.000	0.000	0.000	0.000	0.000	0.000	0.000	0.000
7	Gymnura_poecilura	Gymnura_poecilura	Match	1.000	0.000	0.000	0.000	0.000	0.000	0.000	0.000	0.000	0.000	1.000	0.000	0.000	0.000	0.000	0.000	0.000	0.000	0.000	0.000	0.000	0.000	0.000	0.000	0.000	0.000	0.000	0.000	0.000
8	Neotrygon_orientalis	Neotrygon_orientalis	Match	1.000	0.000	0.000	0.000	0.000	0.000	0.000	0.000	0.000	0.000	0.000	0.000	0.000	0.000	0.000	0.000	0.000	0.000	1.000	0.000	0.000	0.000	0.000	0.000	0.000	0.000	0.000	0.000	0.000
9	Sphyrna_lewini	Sphyrna_lewini	Match	1.000	0.000	0.000	0.000	0.000	0.000	0.000	0.000	0.000	0.000	0.000	0.000	0.000	0.000	0.000	0.000	0.000	0.000	0.000	0.000	0.000	0.000	0.000	0.000	0.000	1.000	0.000	0.000	0.000
10	Sphyrna_lewini	Sphyrna_lewini	Match	1.000	0.000	0.000	0.000	0.000	0.000	0.000	0.000	0.000	0.000	0.000	0.000	0.000	0.000	0.000	0.000	0.000	0.000	0.000	0.000	0.000	0.000	0.000	0.000	0.000	1.000	0.000	0.000	0.000
11	Carcharhinus_sorrah	Stegostoma_fasciatum	Mismatch	0.975	0.000	0.000	0.000	0.000	0.000	0.000	0.025	0.000	0.000	0.000	0.000	0.000	0.000	0.000	0.000	0.000	0.000	0.000	0.000	0.000	0.000	0.000	0.000	0.000	0.000	0.000	0.975	0.000
12	Galeocerdo_cuvier	Galeocerdo_cuvier	Match	1.000	0.000	0.000	0.000	0.000	0.000	0.000	0.000	1.000	0.000	0.000	0.000	0.000	0.000	0.000	0.000	0.000	0.000	0.000	0.000	0.000	0.000	0.000	0.000	0.000	0.000	0.000	0.000	0.000
13	Prionace_glauca	Prionace_glauca	Match	1.000	0.000	0.000	0.000	0.000	0.000	0.000	0.000	0.000	0.000	0.000	0.000	0.000	0.000	0.000	0.000	0.000	0.000	0.000	1.000	0.000	0.000	0.000	0.000	0.000	0.000	0.000	0.000	0.000
14	Rhynchobatus_springer	Rhynchobatus_springer	Match	1.000	0.000	0.000	0.000	0.000	0.000	0.000	0.000	0.000	0.000	0.000	0.000	0.000	0.000	0.000	0.000	0.000	0.000	0.000	0.000	0.000	0.000	0.000	0.000	1.000	0.000	0.000	0.000	0.000
15	Rhynchobatus_springer	Rhynchobatus_springer	Match	1.000	0.000	0.000	0.000	0.000	0.000	0.000	0.000	0.000	0.000	0.000	0.000	0.000	0.000	0.000	0.000	0.000	0.000	0.000	0.000	0.000	0.000	0.000	0.000	1.000	0.000	0.000	0.000	0.000
16	Sphyrna_lewini	Sphyrna_lewini	Match	1.000	0.000	0.000	0.000	0.000	0.000	0.000	0.000	0.000	0.000	0.000	0.000	0.000	0.000	0.000	0.000	0.000	0.000	0.000	0.000	0.000	0.000	0.000	0.000	0.000	1.000	0.000	0.000	0.000
17	Carcharhinus_brevipinn	Carcharhinus_brevipinn	Match	1.000	0.000	0.000	0.000	1.000	0.000	0.000	0.000	0.000	0.000	0.000	0.000	0.000	0.000	0.000	0.000	0.000	0.000	0.000	0.000	0.000	0.000	0.000	0.000	0.000	0.000	0.000	0.000	0.000
18	Carcharhinus_brevipinn	Carcharhinus_brevipinn	Match	1.000	0.000	0.000	0.000	1.000	0.000	0.000	0.000	0.000	0.000	0.000	0.000	0.000	0.000	0.000	0.000	0.000	0.000	0.000	0.000	0.000	0.000	0.000	0.000	0.000	0.000	0.000	0.000	0.000
19	Rhina_ancylostoma	Rhina_ancylostoma	Match	1.000	0.000	0.000	0.000	0.000	0.000	0.000	0.000	0.000	0.000	0.000	0.000	0.000	0.000	0.000	0.000	0.000	0.000	0.000	0.000	0.000	1.000	0.000	0.000	0.000	0.000	0.000	0.000	0.000
20	Alopias_superciliosus	Alopias_superciliosus	Match	1.000	0.000	1.000	0.000	0.000	0.000	0.000	0.000	0.000	0.000	0.000	0.000	0.000	0.000	0.000	0.000	0.000	0.000	0.000	0.000	0.000	0.000	0.000	0.000	0.000	0.000	0.000	0.000	0.000
21	Carcharhinus_longiman	Carcharhinus_longiman	Match	1.000	0.000	0.000	0.000	0.000	0.000	1.000	0.000	0.000	0.000	0.000	0.000	0.000	0.000	0.000	0.000	0.000	0.000	0.000	0.000	0.000	0.000	0.000	0.000	0.000	0.000	0.000	0.000	0.000
22	Himantura_imbricata	Himantura_imbricata	Match	1.000	0.000	0.000	0.000	0.000	0.000	0.000	0.000	0.000	0.000	0.000	1.000	0.000	0.000	0.000	0.000	0.000	0.000	0.000	0.000	0.000	0.000	0.000	0.000	0.000	0.000	0.000	0.000	0.000
23	Isurus_oxyrinchus	Isurus_oxyrinchus	Match	1.000	0.000	0.000	0.000	0.000	0.000	0.000	0.000	0.000	0.000	0.000	0.000	1.000	0.000	0.000	0.000	0.000	0.000	0.000	0.000	0.000	0.000	0.000	0.000	0.000	0.000	0.000	0.000	0.000
24	Isurus_paucus	Isurus_paucus	Match	0.997	0.000	0.000	0.000	0.000	0.000	0.002	0.000	0.000	0.000	0.000	0.000	0.000	0.997	0.000	0.000	0.000	0.000	0.000	0.000	0.000	0.000	0.000	0.000	0.000	0.000	0.000	0.000	0.000
25	Rhynchobatus_australia	Rhynchobatus_australia	Match	1.000	0.000	0.000	0.000	0.000	0.000	0.000	0.000	0.000	0.000	0.000	0.000	0.000	0.000	0.000	0.000	0.000	0.000	0.000	0.000	0.000	0.000	1.000	0.000	0.000	0.000	0.000	0.000	0.000
26	Sphyrna_mokarran	Glaucostegus_typus	Mismatch	0.802	0.002	0.000	0.000	0.001	0.000	0.190	0.000	0.000	0.802	0.000	0.001	0.000	0.000	0.000	0.000	0.000	0.000	0.000	0.000	0.001	0.001	0.001	0.000	0.000	0.000	0.000	0.000	0.000
27	Telatrygon_zugei	Telatrygon_zugei	Match	1.000	0.000	0.000	0.000	0.000	0.000	0.000	0.000	0.000	0.000	0.000	0.000	0.000	0.000	0.000	0.000	0.000	0.000	0.000	0.000	0.000	0.000	0.000	0.000	0.000	0.000	0.000	0.000	1.000
28	Carcharhinus_brevipinn	Carcharhinus_brevipinn	Match	1.000	0.000	0.000	0.000	1.000	0.000	0.000	0.000	0.000	0.000	0.000	0.000	0.000	0.000	0.000	0.000	0.000	0.000	0.000	0.000	0.000	0.000	0.000	0.000	0.000	0.000	0.000	0.000	0.000
29	Carcharhinus_brevipinn	Carcharhinus_brevipinn	Match	1.000	0.000	0.000	0.000	1.000	0.000	0.000	0.000	0.000	0.000	0.000	0.000	0.000	0.000	0.000	0.000	0.000	0.000	0.000	0.000	0.000	0.000	0.000	0.000	0.000	0.000	0.000	0.000	0.000
30	Carcharhinus_brevipinn	Carcharhinus_brevipinn	Match	1.000	0.000	0.000	0.000	1.000	0.000	0.000	0.000	0.000	0.000	0.000	0.000	0.000	0.000	0.000	0.000	0.000	0.000	0.000	0.000	0.000	0.000	0.000	0.000	0.000	0.000	0.000	0.000	0.000
31	Carcharhinus_falciformi	Carcharhinus_falciformi	Match	0.995	0.000	0.000	0.000	0.005	0.995	0.000	0.000	0.000	0.000	0.000	0.000	0.000	0.000	0.000	0.000	0.000	0.000	0.000	0.000	0.000	0.000	0.000	0.000	0.000	0.000	0.000	0.000	0.000
32	Galeocerdo_cuvier	Glaucostegus_typus	Mismatch	1.000	0.000	0.000	0.000	0.000	0.000	0.000	0.000	0.000	1.000	0.000	0.000	0.000	0.000	0.000	0.000	0.000	0.000	0.000	0.000	0.000	0.000	0.000	0.000	0.000	0.000	0.000	0.000	0.000
33	Galeocerdo_cuvier	Glaucostegus_typus	Mismatch	0.923	0.000	0.000	0.000	0.000	0.000	0.000	0.000	0.050	0.923	0.000	0.000	0.026	0.000	0.000	0.000	0.000	0.000	0.000	0.000	0.000	0.000	0.000	0.000	0.000	0.000	0.000	0.000	0.000
34	Galeocerdo_cuvier	Glaucostegus_typus	Mismatch	1.000	0.000	0.000	0.000	0.000	0.000	0.000	0.000	0.000	1.000	0.000	0.000	0.000	0.000	0.000	0.000	0.000	0.000	0.000	0.000	0.000	0.000	0.000	0.000	0.000	0.000	0.000	0.000	0.000
35	Galeocerdo_cuvier	Glaucostegus_typus	Mismatch	1.000	0.000	0.000	0.000	0.000	0.000	0.000	0.000	0.000	1.000	0.000	0.000	0.000	0.000	0.000	0.000	0.000	0.000	0.000	0.000	0.000	0.000	0.000	0.000	0.000	0.000	0.000	0.000	0.000
36	Glaucostegus_typus	Glaucostegus_typus	Match	1.000	0.000	0.000	0.000	0.000	0.000	0.000	0.000	0.000	1.000	0.000	0.000	0.000	0.000	0.000	0.000	0.000	0.000	0.000	0.000	0.000	0.000	0.000	0.000	0.000	0.000	0.000	0.000	0.000
37	Glaucostegus_typus	Glaucostegus_typus	Match	1.000	0.000	0.000	0.000	0.000	0.000	0.000	0.000	0.000	1.000	0.000	0.000	0.000	0.000	0.000	0.000	0.000	0.000	0.000	0.000	0.000	0.000	0.000	0.000	0.000	0.000	0.000	0.000	0.000
38	Glaucostegus_typus	Glaucostegus_typus	Match	1.000	0.000	0.000	0.000	0.000	0.000	0.000	0.000	0.000	1.000	0.000	0.000	0.000	0.000	0.000	0.000	0.000	0.000	0.000	0.000	0.000	0.000	0.000	0.000	0.000	0.000	0.000	0.000	0.000
39	Glaucostegus_typus	Glaucostegus_typus	Match	1.000	0.000	0.000	0.000	0.000	0.000	0.000	0.000	0.000	1.000	0.000	0.000	0.000	0.000	0.000	0.000	0.000	0.000	0.000	0.000	0.000	0.000	0.000	0.000	0.000	0.000	0.000	0.000	0.000
40	Prionace_glauca	Prionace_glauca	Match	0.929	0.000	0.000	0.000	0.071	0.000	0.000	0.000	0.000	0.000	0.000	0.000	0.000	0.000	0.000	0.000	0.000	0.000	0.000	0.929	0.000	0.000	0.000	0.000	0.000	0.000	0.000	0.000	0.000
41	Prionace_glauca	Carcharhinus_brevipinn	Mismatch	0.878	0.000	0.000	0.000	0.878	0.000	0.000	0.000	0.000	0.000	0.000	0.000	0.000	0.000	0.000	0.000	0.000	0.000	0.000	0.122	0.000	0.000	0.000	0.000	0.000	0.000	0.000	0.000	0.000
42	Prionace_glauca	Prionace_glauca	Match	0.663	0.000	0.000	0.000	0.337	0.000	0.000	0.000	0.000	0.000	0.000	0.000	0.000	0.000	0.000	0.000	0.000	0.000	0.000	0.663	0.000	0.000	0.000	0.000	0.000	0.000	0.000	0.000	0.000
43	Prionace_glauca	Prionace_glauca	Match	0.735	0.000	0.000	0.000	0.192	0.073	0.000	0.000	0.000	0.000	0.000	0.000	0.000	0.000	0.000	0.000	0.000	0.000	0.000	0.735	0.000	0.000	0.000	0.000	0.000	0.000	0.000	0.000	0.000
44	Rhynchobatus_laevis	Isurus_paucus	Mismatch	0.740	0.003	0.001	0.000	0.000	0.000	0.000	0.000	0.000	0.006	0.000	0.000	0.000	0.740	0.000	0.003	0.004	0.001	0.000	0.000	0.004	0.014	0.077	0.136	0.009	0.000	0.000	0.001	0.000
45	Rhynchobatus_laevis	Rhynchobatus_laevis	Match	1.000	0.000	0.000	0.000	0.000	0.000	0.000	0.000	0.000	0.000	0.000	0.000	0.000	0.000	0.000	0.000	0.000	0.000	0.000	0.000	0.000	0.000	0.000	1.000	0.000	0.000	0.000	0.000	0.000
46	Rhynchobatus_springer	Pristis_pristis	Mismatch	0.583	0.000	0.369	0.002	0.000	0.000	0.000	0.000	0.000	0.002	0.001	0.003	0.000	0.001	0.000	0.003	0.024	0.005	0.000	0.002	0.583	0.003	0.000	0.001	0.000	0.000	0.000	0.000	0.000
47	Sphyrna_lewini	Mobula_tarapacana	Mismatch	0.724	0.000	0.000	0.000	0.000	0.000	0.000	0.000	0.000	0.000	0.000	0.000	0.000	0.000	0.000	0.000	0.000	0.724	0.000	0.000	0.000	0.000	0.000	0.000	0.000	0.276	0.000	0.000	0.000
48	Sphyrna_lewini	Sphyrna_lewini	Match	0.785	0.000	0.000	0.009	0.000	0.005	0.000	0.004	0.000	0.001	0.000	0.000	0.001	0.000	0.000	0.021	0.009	0.156	0.000	0.000	0.000	0.000	0.000	0.000	0.000	0.785	0.005	0.003	0.000
49	Sphyrna_lewini	Glaucostegus_typus	Mismatch	0.914	0.000	0.000	0.000	0.000	0.000	0.000	0.000	0.000	0.914	0.000	0.000	0.001	0.000	0.000	0.000	0.001	0.000	0.000	0.000	0.000	0.000	0.000	0.000	0.001	0.083	0.000	0.000	0.000
50	Sphyrna_lewini	Sphyrna_lewini	Match	0.987	0.000	0.000	0.000	0.000	0.008	0.000	0.000	0.000	0.001	0.000	0.000	0.000	0.000	0.000	0.000	0.000	0.002	0.001	0.000	0.000	0.000	0.000	0.000	0.000	0.987	0.000	0.000	0.000
51	Alopias_superciliosus	Alopias_superciliosus	Match	0.608	0.000	0.608	0.000	0.000	0.000	0.000	0.000	0.000	0.000	0.000	0.000	0.000	0.000	0.000	0.000	0.000	0.000	0.000	0.000	0.000	0.000	0.000	0.392	0.000	0.000	0.000	0.000	0.000
52	Carcharhinus_longiman	Glaucostegus_typus	Mismatch	1.000	0.000	0.000	0.000	0.000	0.000	0.000	0.000	0.000	1.000	0.000	0.000	0.000	0.000	0.000	0.000	0.000	0.000	0.000	0.000	0.000	0.000	0.000	0.000	0.000	0.000	0.000	0.000	0.000
53	Carcharhinus_longiman	Glaucostegus_typus	Mismatch	1.000	0.000	0.000	0.000	0.000	0.000	0.000	0.000	0.000	1.000	0.000	0.000	0.000	0.000	0.000	0.000	0.000	0.000	0.000	0.000	0.000	0.000	0.000	0.000	0.000	0.000	0.000	0.000	0.000
54	Carcharhinus_longiman	Glaucostegus_typus	Mismatch	1.000	0.000	0.000	0.000	0.000	0.000	0.000	0.000	0.000	1.000	0.000	0.000	0.000	0.000	0.000	0.000	0.000	0.000	0.000	0.000	0.000	0.000	0.000	0.000	0.000	0.000	0.000	0.000	0.000
55	Isurus_oxyrinchus	Isurus_oxyrinchus	Match	1.000	0.000	0.000	0.000	0.000	0.000	0.000	0.000	0.000	0.000	0.000	0.000	1.000	0.000	0.000	0.000	0.000	0.000	0.000	0.000	0.000	0.000	0.000	0.000	0.000	0.000	0.000	0.000	0.000
56	Sphyrna_mokarran	Sphyrna_mokarran	Match	0.973	0.000	0.000	0.000	0.000	0.000	0.015	0.000	0.000	0.001	0.000	0.000	0.000	0.001	0.003	0.007	0.000	0.000	0.000	0.000	0.000	0.000	0.000	0.000	0.000	0.000	0.973	0.000	0.000
57	Stegostoma_fasciatum	Stegostoma_fasciatum	Match	0.523	0.000	0.000	0.000	0.000	0.000	0.000	0.477	0.000	0.000	0.000	0.000	0.000	0.000	0.000	0.000	0.000	0.000	0.000	0.000	0.000	0.000	0.000	0.000	0.000	0.000	0.000	0.523	0.000
58	Alopias_pelagicus	Alopias_pelagicus	Match	1.000	1.000	0.000	0.000	0.000	0.000	0.000	0.000	0.000	0.000	0.000	0.000	0.000	0.000	0.000	0.000	0.000	0.000	0.000	0.000	0.000	0.000	0.000	0.000	0.000	0.000	0.000	0.000	0.000
59	Anoxypristis_cuspidata	Anoxypristis_cuspidata	Match	1.000	0.000	0.000	1.000	0.000	0.000	0.000	0.000	0.000	0.000	0.000	0.000	0.000	0.000	0.000	0.000	0.000	0.000	0.000	0.000	0.000	0.000	0.000	0.000	0.000	0.000	0.000	0.000	0.000
60	Lamna_nasus	Lamna_nasus	Match	1.000	0.000	0.000	0.000	0.000	0.000	0.000	0.000	0.000	0.000	0.000	0.000	0.000	0.000	1.000	0.000	0.000	0.000	0.000	0.000	0.000	0.000	0.000	0.000	0.000	0.000	0.000	0.000	0.000
61	Lamna_nasus	Lamna_nasus	Match	1.000	0.000	0.000	0.000	0.000	0.000	0.000	0.000	0.000	0.000	0.000	0.000	0.000	0.000	1.000	0.000	0.000	0.000	0.000	0.000	0.000	0.000	0.000	0.000	0.000	0.000	0.000	0.000	0.000
62	Mobula_birostris	Mobula_birostris	Match	1.000	0.000	0.000	0.000	0.000	0.000	0.000	0.000	0.000	0.000	0.000	0.000	0.000	0.000	0.000	1.000	0.000	0.000	0.000	0.000	0.000	0.000	0.000	0.000	0.000	0.000	0.000	0.000	0.000
63	Mobula_mobular	Mobula_mobular	Match	1.000	0.000	0.000	0.000	0.000	0.000	0.000	0.000	0.000	0.000	0.000	0.000	0.000	0.000	0.000	0.000	1.000	0.000	0.000	0.000	0.000	0.000	0.000	0.000	0.000	0.000	0.000	0.000	0.000
64	Mobula_tarapacana	Mobula_tarapacana	Match	1.000	0.000	0.000	0.000	0.000	0.000	0.000	0.000	0.000	0.000	0.000	0.000	0.000	0.000	0.000	0.000	0.000	1.000	0.000	0.000	0.000	0.000	0.000	0.000	0.000	0.000	0.000	0.000	0.000
65	Pristis_pristis	Pristis_pristis	Match	1.000	0.000	0.000	0.000	0.000	0.000	0.000	0.000	0.000	0.000	0.000	0.000	0.000	0.000	0.000	0.000	0.000	0.000	0.000	0.000	1.000	0.000	0.000	0.000	0.000	0.000	0.000	0.000	0.000
66	Carcharhinus_falciformi	Carcharhinus_falciformi	Match	1.000	0.000	0.000	0.000	0.000	1.000	0.000	0.000	0.000	0.000	0.000	0.000	0.000	0.000	0.000	0.000	0.000	0.000	0.000	0.000	0.000	0.000	0.000	0.000	0.000	0.000	0.000	0.000	0.000
67	Carcharhinus_falciformi	Carcharhinus_falciformi	Match	1.000	0.000	0.000	0.000	0.000	1.000	0.000	0.000	0.000	0.000	0.000	0.000	0.000	0.000	0.000	0.000	0.000	0.000	0.000	0.000	0.000	0.000	0.000	0.000	0.000	0.000	0.000	0.000	0.000
68	Carcharhinus_falciformi	Carcharhinus_falciformi	Match	1.000	0.000	0.000	0.000	0.000	1.000	0.000	0.000	0.000	0.000	0.000	0.000	0.000	0.000	0.000	0.000	0.000	0.000	0.000	0.000	0.000	0.000	0.000	0.000	0.000	0.000	0.000	0.000	0.000

Table S.6. Initial value of hyper-parameters in searching for the best deep learning model using grid search method

Parameters	Definition	Value
activation	The activation function of learning model	"Rectifier", "Maxout", "Tanh", "RectifierWithDropout", "MaxoutWithDropout" and "TanhWithDropout"
hidden	Number of learning layers	[100, 100, 100], [200, 200, 200] and [500, 500, 500]
epochs	Number of times to iterate (stream) the dataset	50, 100, 200, 300 and 500
rho	The adaptive learning rate time decay factor	0.9, 0.95, 0.99 and 0.999
epsilon	The adaptive learning rate time smoothing factor to avoid dividing by zero	1e-10, 1e-8, 1e-6 and 1e-4
input_dropout_ratio	The input layer dropout ratio to improve generalisation. Suggested values are 0.1 or 0.2	0, 0.1 and 0.2
l1	The L1 regularization to add stability and improve generalisation	0, 0.00001 and 0.0001
l2	The L2 regularization to add stability and improve generalisation	0, 0.00001 and 0.0001
max_w2	The constraint for the squared sum of the incoming weights per unit	10, 100, 1000 and 3.4028235e+38

Table S.7. Stopping criteria in searching the best deep learning model

Criteria	Definition	Value
strategy	strategy to perform a random search of all the combinations of your hyperparameters	RandomDiscrete
max_models	The maximum number of generated models	100,000
max_runtime_secs	The maximum run time in second	43,200 seconds (12 hours)
stopping_tolerance	Stop if MSE hasn't improved by the value	0.001
stopping_rounds	Number of models to compare MSE improvement	20
seed	Seed number to control randomness	1234
